# Do we need standardized postoperative radiographs after locking plate fixation of distal radius fractures – A retrospective analysis of 664 patients

**DOI:** 10.1007/s00402-024-05631-9

**Published:** 2024-12-12

**Authors:** V. Ketter, C. Kiehne, M. Frink, R. Aigner, S. Ruchholtz, J. Lenz

**Affiliations:** 1 Center for Orthopaedics and Trauma Surgery, Philipps-University Marburg; University Hospital Giessen and Marburg GmbH, Location Marburg, Baldingerstraße, D-35043 Marburg, Germany; 2 Department of Dermatology, Allergology and Venereology, University Hospital Schleswig-Holstein, Location, Lübeck, Germany; 3 Orthopraxis Kaufbeuren, Kaufbeuren, Germany

**Keywords:** Distal radius fracture, Radiation, Postoperative radiograph

## Abstract

**Introduction:**

After surgical treatment of fractures of the distal radius, radiographs in 2 planes are routinely performed postoperatively as a standard procedure to verify anatomic reduction and implant positioning. However, the postoperative radiological examinations rarely has a consequence. Therefore, the purpose of this study was to evaluate the frequency of treatment plan changes based on standardized postoperative radiographs. Secondarily, abnormalities, already being present in the intraoperative radiographs, were examined.

**Methods:**

Between 2015 and 2019 a total of 664 consecutive patients who underwent open reduction and internal fixation of a distal radius were evaluated in a retrospective study.

**Results:**

The median age was 60 (range 92 − 16 years). Overall, a treatment plan change was detected in 20 patients. After standardized postoperative radiographs a CT scan was performed in 16 patients and 14 patients underwent early operative revision; in only four of these cases, the revision could be attributed to the postoperative radiograph.

**Conclusion:**

Considering the low incidence of treatment plan changes, routinely performed radiographs after surgical treatment of distal radius fractures must be critically questioned.

## Background

Fractures of the distal radius are among the most common fractures in humans, accounting for approximately 15% of all extremity fractures [1]. In 2013, a large epidemiological study from Scandinavia calculated an annual incidence of 31/10 000 people [2]. Open reduction and internal fixation is the standard procedure for the treatment of displaced distal radius fractures [3]. Postoperative radiographs in two standard planes are usually taken to check and document the result of the operation [4]. In addition, intraoperative fluoroscopy has been shown to have comparable diagnostic reliability to postoperative radiographic imaging [5].

Due to radiation exposure, additional costs of postoperative imaging, and the upcoming era of ambulatory care the question arises, whether routine postoperative radiography is necessary. The aim of this study was to examine how often postoperative radiographs led to revision surgery.

## Materials and methods

### Patients

This retrospective study included all consecutive patients who underwent surgical fixation of a distal radius fracture with locking plates between January 2015 and December 2019.

The ethics committee of the Department of Medicine at Philipps University Marburg (AZ ek_mr_26_10_2020_frink) gave a positive ethics vote for the data collection.

The following exclusion criteria were applied:

1. Age < 18 years.
2. Missing intraoperative fluoroscopy or postoperative radiographs.
3. Surgical treatment in another hospital.
4. Other surgical procedures than locking plate fixation.

The diagnosis of the fracture was based on plain radiographs. In case of complex intraarticular fractures, a pre-operative CT scan was performed to gain an exact understanding of the fracture pattern and for pre-operative planning. Fracture reduction - if necessary - was performed under dorsal local anaesthesia in the fracture gap and casts were applied in the emergency department. Afterwards, surgical treatment was scheduled, and patients remained ambulatory until surgery, which was performed after a median 4 days (range 0–36 days). Palmar plate fixation of the distal radius fracture was performed via Henry’s approach [6]. In case of fractures with severe dorsal defects, a dorsal approach and dorsal plate positioning was performed. The choice of the approach was dependent on the surgeon’s decision. Intraoperative fluoroscopy was used in all patients to control adequate reduction and correct plate positioning. The two standard planes frontal, and lateral view were performed, each plane taken by holding the forearm in an angle of 15 to 20 degrees from the table. The intraoperative radiographs were stored digitally and were available for digital image processing. Antibiotic prophylaxis was performed with second generation cephalosporin. A protective splint was utilized for two days; afterwards, free range of motion was allowed without any weight bearing for six weeks.

The main goal of this study was to determine the influence of postoperative radiograph imaging on the postoperative treatment regime.

In addition to that, abnormalities (mispositioning of the plate, intraarticular screw positioning, incomplete reduction of the fracture, periimplant fractures) which were detected in postoperative radiographs, were retrospectively analyzed, whether a detection of the abnormality could have been possible in the intraoperative imaging as well.

### Evaluation of the data

Demographics and patient parameters age, gender, trauma mechanism, fracture localization, accompanying injuries, and co-morbidities [7] were recorded. Further clinical data like fracture classification [8], operation time, operating surgeon, in-hospital time, position of the plate (palmar/dorsal), were also documented as well as complications related to surgery.

Subsequently, all changes in treatment and revisions were analyzed. For that purpose, additional CT scans, which we do not perform routinely or a revision surgery due to postoperative imaging were recorded. Furthermore, it was analyzed by two senior trauma surgeons (M.F., R.A.) whether the abnormalities like mispositioning of the plate, intraarticular screw positioning, incomplete reduction of the fracture and peri-implant fractures, that led to the change in treatment (additional CT-scan or early revision), had already been visible in intraoperative imaging. Data were collected and a pseudonymization was performed. Descriptive statistics were used to describe clinical characteristics, complications and outcomes. Data were presented as median and range values.

## Results

A total of 664 patients were included. The median age was 60 years (range 16–92 years) and the median hospital length of stay was 3 days (range 0–48 days). 471 patients were female (70%). The median time of surgery was 58 min (range 20–200 min). Fracture types, as categorized by the AO classification, are shown in Table 1. The majority of patients (84% (*n* = 560)) were treated with palmar locking plates.

**Table 1:** Baseline characteristics

N = 664	
Age (years)	median 60, range 16–92 years
Gender	471 female, 194 male
Classification	
- A1	0
- A2	117
- A3	92
- B1	11
- B2	5
- B3	7
- C1	61
- C2	180
- C3	185
- Other fractures	6
Plate position (palmar/dorsal)	560 palmar, 104 dorsal
Associated fracture (yes/no)	423 yes (244 PSU-Avulsion), 248 no

423 patients had concomitant fractures, of which 244 were avulsion fractures of the *processus styloideus ulnae* (PSU).

In addition to the pathologies detected by radiographics, irritations of the median nerve (*n* = 20), infections (*n* = 2) and vascular injuries (*n* = 1) were recorded as perioperative complications.

**Fig. 1:**
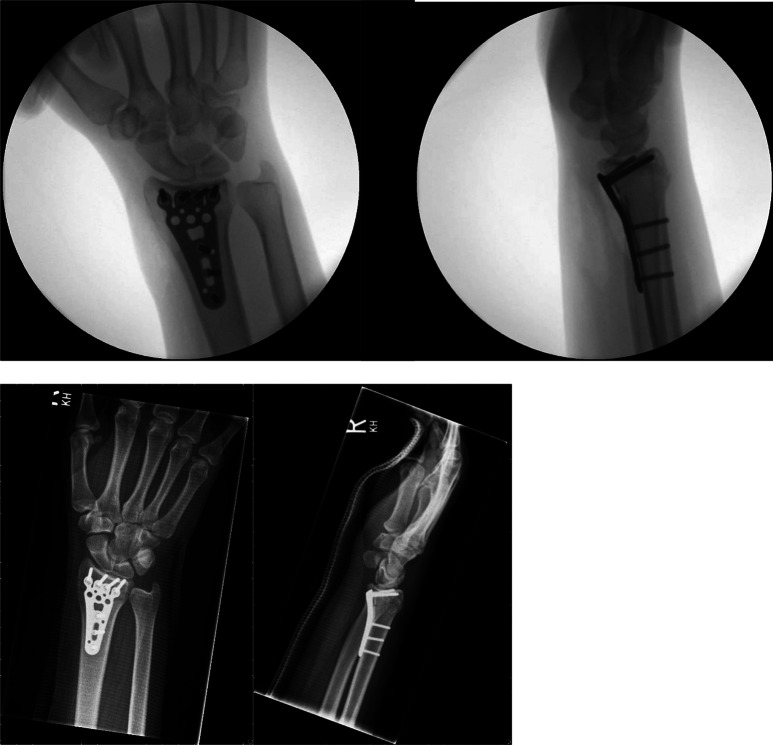
Patient 1: intraoperative and postoperative imaging without abormalities

**Fig. 2:**
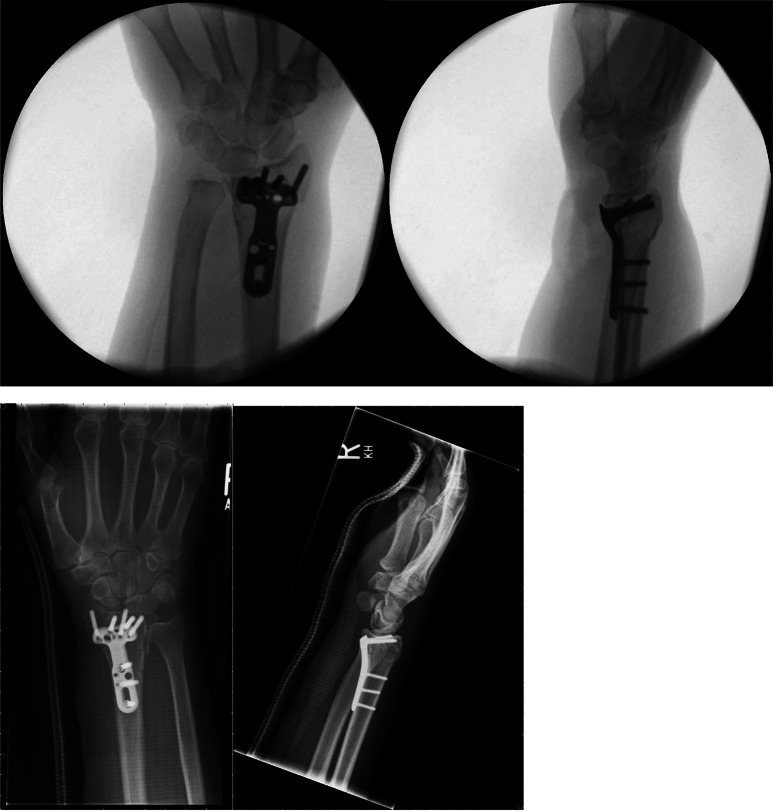
Patient 2: intraoperative imaging without abormalities, postoperative radiographs with suspected intraarticular screw positioning

The treatment was influenced by postoperative radiographs in 16 patients (2%). Figure 1 shows intraoperative and postoperative radiographs without abnormalities. In Figure 2 an example for a postoperative radiograph with an abnormality is shown. All of them had an additional CT scan, which was performed as a consequence of postoperative standard radiographs, to evaluate implant positioning or to detect an inadequate fracture reposition or intraarticular screw positioning. Five of these patients (31%) underwent early revision surgery as a consequence of the CT scan.

Most common reasons for revision surgery after CT scan or standard imaging (radiographs) were poor reduction with fracture malpositioning, such as intra-articular step formation (*n* = 5, 75%), unfixed fracture fragments (*n* = 1, 12%), peri-implant fractures (*n* = 1, 12%), a malposition of the implant (*n* = 1, 12%), as well as a non-visualized joint space (*n* = 1, 12%).

**Fig. 3:**
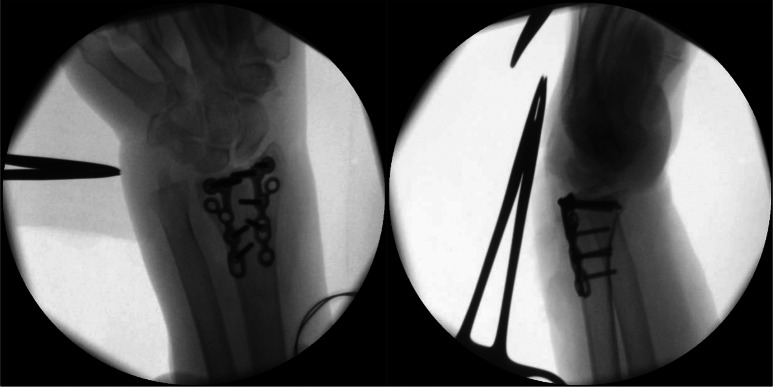
Patient 3: abnormalities already seen in the intraoperative imaging

In 14 cases (2%), revision surgery was indicated within the first week after the index operation. This could be attributed to postoperative imaging in 9 cases (consequence of postoperative radiographs: *n* = 4, consequence of postoperative CT-scan: *n* = 5). An example is shown in Fig. 3.

In five patients, revision surgery was not indicated due to postoperative imaging but due to clinical symptoms. Reasons were an irritation of the median nerve in four patients, of whom one patient also had a vascular injury. Another patient developed a wound infection and underwent early revision surgery with debridement and implant retaining.

The reasons for the revisions are shown in Table 2.

**Table 2:** Reasons for revisions

Reasons for revision indicated due postoperative imaging	Patients
Mispositioning of the plate, intraarticular screw positioning, fracture not completely reduced	8
peri-implant fractures	1
*Reasons for revision not indicated due postoperative imaging*	
Carpal tunnel syndrome	3
Carpal tunnel syndrome & vascular injury	1
Wound infection	1

## Discussion

This study showed a low rate of treatment changes after standardized postoperative radiographs in patients with fractures of the distal radius who underwent surgery. In an era of increasing ambulatory care and reduced radiology capacity, the need for postoperative radiographs must be critically discussed.

The question of the need for additional imaging after surgical treatment of various fractures has recently been critically questioned following the publication of small collectives with controversial results [9–12]. Oehme et al. [9] concluded that postoperative radiographs could improve quality of care, while Sharma et al. [13] questioned the value of postoperative imaging since changes in postoperative treatment pathways are rarely detected.

In this study, postoperative radiographs, which were taken before discharge, didn’t have any consequences in 98% of the cases.

The majority of pathologies could have been detected in intraoperative radiographs.

In only 16 patients (2%), the treatment algorithm was changed by performing an additional CT scan as a result of the postoperative radiographs.

14 patients (2%) underwent early revision surgery.

In nine patients, revision surgery could be attributed to postoperative imaging, either standardized postoperative radiographs (four patients) or additional CT scans (five patients). In two patients an invisible joint space was already visible on the intraoperative radiographs.

It is difficult to answer why the surgeon did not react immediately during surgery. It is evident that the identification of abnormalities observed during surgery is facilitated by the postoperative radiograph, particularly when the surgical procedure has been revised.

Oehme et al. [12] included 316 patients requiring surgery for a distal radius fracture or an ankle fracture in a prospective, randomized, controlled, non-blinded trial. Patients in the control group underwent postoperative radiographs, while patients in the interventional group did not. Primary endpoint was a change in the treatment plan, defined as additional imaging or a revision surgery. A change in treatment plan was identified in only 3% of patients and the frequency of changes was comparable in both groups. Postoperative imaging was obtained in 3% of all patients. The results of this study are comparable to the present results (changes in treatment plan: 2%), however Oehme et al. [12] investigated not only distal radius fractures but ankle fractures as well. Based on their results, intraoperative fluoroscopy seems to be superior to postoperative radiograph imaging, especially in radius fractures, as it allows dynamic adjustment with a clear visualization of the joint to ensure adequate screw positioning outside the articular gap. Moreover, Oehme at al. [12] have shown, that additional postoperative radiographs in patients with distal radius and ankle fractures do not improve patients’ pain, mobility or clinical outcome.

Regarding to the findings of these studies minimizing the number of postoperative radiographs could have a positive impact by reducing costs and probably become more important in an upcoming era of ambulatory care.

Several studies confirmed that intraoperative radiographics could document the quality of reduction and implant positioning even in a reproducible way [14]. Harish et al. [15] showed no differences between intraoperative and postoperative imaging in 83.3% of patients with ankle fractures. Similar results were published by Haddad et al. [14], who compared intraoperative imaging with postoperative radiographs after closed reduction and internal fixation of 80 patients with proximal femoral fractures. The postoperative radiographs provided no additional information.

Based on the present findings postoperative images had not a higher quality than the intraoperative radiographs in most of the cases, which was attributed to limited mobility and a decreased visualization due to pre-existing splints, which haven’t been removed before imaging. This led to difficulties in evaluating the joint space of the wrist. However, adequate intraoperative imaging could be challenging as well, especially in fractures with severe dorsal defects. The two patients, in whom abnormalities were detected by postoperative imaging, but with a joint gap already visible in the intraoperative radiographs, had fracture with severe dorsal defects.

A prerequisite for renouncing with postoperative radiograph documentation is, of course, the possibility of long-term storage of intraoperative imaging. At best, this is done by transferring the intraoperative image data to the hospital’s information system. However, this is increasingly becoming a standard in hospitals.

## Limitations

The findings of the present study are limited by several factors. First of all, this is a retrospective study design.

Furthermore, not every hospital has the same ability to archive intraoperative images.

It should be noted that some postoperative radiographs were taken on the second postoperative day in a splint, which makes it challenging to assess the image. This represents a further limitation of the study.As long as there is no standard protocol and no possibility for digital long-term storage, postoperative radiographs should not be dispensed with.

Moreover, data is strongly dependent on documentation quality, although patients’ records were carefully reviewed.

## Conclusion

Based on the results of this study, additional postoperative standard radiographs of the wrist after open reduction and internal fixation of a distal radius fracture must be critically scrutinised if a correct intraoperative radiograph was taken with an image converter and digitally archived, especially at a time of increasing financial pressure in the healthcare sector and the upcoming era of ambulatory care.

Therefore, standardized protocols for intraoperative imaging are needed and surgeons need to be aware of them.

Otherwise, postoperative radiographs should not be dispensed with.

## Data Availability

The data that support the findings of this study are available on request from the corresponding author.
